# Correlation between gut microbiota dysbiosis, metabolic syndrome and breast cancer

**DOI:** 10.1038/s41598-025-89801-8

**Published:** 2025-02-24

**Authors:** Eslam M. Abdelqader, Walaa S. Mahmoud, Hassan M. Gebreel, Mahmoud M. Kamel, Mohammed Abu-Elghait

**Affiliations:** 1 Department of Microbiology, Faculty of Science, Ain Shams University, Cairo, Egypt; 2 Biological Anthropology Department National Research Centre, Dokki, Giza Egypt; 3 Clinical Pathology Development, National Cancer Institute Cairo University, Cairo, Egypt; 4 Laboratory Development Bahyea Centre for Early Detection and Cancer Treatment, Cairo, Egypt; 5 Department of Botany and Microbiology, Faculty of Science, Al-Azhar University, Cairo, 11884 Egypt

**Keywords:** Microbiology, Clinical microbiology

## Abstract

Breast cancer is a widespread cancer with a high death rate globally. The incidence of breast cancer is expected to increase, particularly in low and middle-income countries due to environmental factors and lifestyle changes. Several risk factors, such as age, family history, hormonal and reproductive factors, have been identified to influence breast cancer development. Metabolic syndrome, is a metabolic disorder that has also been linked to breast cancer risk. The gut microbiome has been suggested as one of the environmental factors leading to breast cancer. The human microbiome is mainly colonized in the intestine by various bacterial species, including *Lactobacillus*, *Bifidobacterium*, and *Streptococcus* and protect the host against pathogenic microorganisms and regulate the immune system. This study included 50 female breast cancer patients and 50 healthy controls with matched ages. Stool fresh samples were taken from test and control groups and stored at − 20 °C until further investigations. DNA of the bacteria in stool samples was extracted using reverse transcription-quantitative polymerase chain reaction to check for the bacterial 16s rRNA gene. The exclusion criteria included other malignancies, recent intestinal surgery, infectious diarrhea, prolonged use of antibiotics, substance addiction, and pregnancy or lactation. Our findings exhibited that breast cancer patients had a higher incidence of metabolic syndrome (60%) compared to cancer-free controls (40%). Furthermore, breast cancer patients had significantly lower *Bifidobacterium* and *Lactobacillus* counts than the controls. No significant difference was found in *Streptococcus* counts between groups. These findings support the relationship between breast cancer and metabolic syndrome and suggest the potential involvement of *Lactobacillus* and *Bifidobacterium* in breast cancer pathophysiology. Our study supports the relation between breast cancer and disorder of metabolic syndrome and suggests the potential involvement of *Lactobacillus* and *Bifidobacterium* in breast cancer pathophysiology. Further research is necessary to investigate the complex interactions between genes, the environment, and the gut microbiome in breast cancer development. Understanding these interactions could lead to the progress of novel strategies for breast cancer prevention and treatment.

## Introduction

Breast cancer has been documented in written medicine since ancient times, with reports and illustrations originating from ancient Egypt. A papyrus dating back to 3000–2500 BC, presumably written by the Egyptian physician Imhotep, provided accurate descriptions of breast tumors, which were considered untreatable and characterized as “cool to touch, bulging, and spread all over the breast”^1,2^. Currently, Breast cancer is currently the most frequently diagnosed cancer worldwide. In 2020, it accounted for approximately 2.3 million cases globally and was the fifth leading cause of cancer-related deaths^3,4^. The number of newly diagnosed cases is expected to increase to 2.7 million annually by 2030, with deaths reaching 0.87 million^5^.

Breast cancer is the most prevalent malignant disease among women in Egypt, with the second highest mortality rate in 2020^4,6^. In low and middle-income countries, breast cancer cases is expected to rise due to various environmental factors affecting young generations, better cancer detection programs, and westernizing lifestyles^3,6,7^. Age, family history, hormonal and reproductive factors are the most important risk factors for breast cancer development^8^. Raising awareness of these risk factors is crucial for motivating women to participate in the prevention, early detection, and management of the disease^9^.

Age, family history, hormonal and reproductive factors are the most important risk factors for breast cancer development^8^. Raising awareness of these risk factors is crucial for motivating women to participate in the prevention, early detection, and management of the disease^9^. The incidence and mortality of breast cancer increase with age. Globally, breast cancer peaks around age 60. The median age at diagnosis in Egypt is younger, at around 46 to 54 years old^10^. However, breast cancer can occur in women of all ages. Race and ethnicity also play a role in breast cancer risk. Caucasian women are more likely to develop breast cancer than African-American women, but African-American women are more likely to die from^1^ it at any age. Asian, Hispanic, and Native American women have a lower risk of developing and dying from breast cancer. Certain benign breast conditions can also increase breast cancer risk. Women with dense breasts on a mammogram have a higher risk of breast cancer. Lobular carcinoma in situ (LCIS) is another condition that increases breast cancer risk^11^.

Lifestyle factors and socioeconomic status significantly influence breast cancer risk and outcomes. Lifestyle choices such as alcohol consumption, physical inactivity, and poor diet are associated with an increased risk of breast cancer. For instance, alcohol intake has been linked to higher breast cancer risk, while regular physical activity and maintaining a healthy weight can reduce this risk^12^. Socioeconomic status also plays a crucial role. Women with higher socioeconomic often exhibit increased breast cancer incidence, potentially due to reproductive behaviors and access to screening. Conversely, lower socioeconomic is associated with later-stage diagnoses and poorer survival rates, possibly due to limited access to healthcare resources and disparities in treatment^13^.

Metabolic syndrome (MetS) is a metabolic disorder illustrated by the presence of at least three characteristics from: fasting blood glucose ≥ 100 mg/dL, waist circumference ≥ 80 cm in women, triglyceride levels ≥ 150 mg/dL, HDL cholesterol < 50 mg/dL in women, and blood pressure ≥ 130/85 mmHg^14–16^. MetS can increase breast cancer risk through modifying different hormonal pathways, including those related to insulin, estrogen, cytokines, and growth factors^17–19^.

Breast cancer development is influenced by complex interactions between genes and the environment. While rare genetic mutations and exposure to the environment can lead to breast cancer, only a small percentage of cases develop under these well-known conditions^20^. Thus, there may be other undefined pathways leading to or promoting breast cancer. The bacterial communities within the host, particularly those in the gastrointestinal tract (GI), could be one of the environmental factors involved in breast cancer^21,22^.

The human microbiome consists of 100 trillion bacteria, protozoa, fungi, and viruses, which play a crucial role in health and disease through countless biological processes^23–25^. The intestine is the most colonized body site, with 500–1000 species of bacteria belonging to four main phyla: Firmicutes, Bacteroidetes, Actinobacteria, and Proteobacteria^25–28^. Lactobacilli and Bifidobacteria, two genera of gut bacteria, produce acids such as lactic acid, short-chain fatty acids, and bacteriocins. Short-chain fatty acids stimulate mucus production, prevent inflammation, and increase total and pathogen-specific mucosal IgA, making them important for health^29,30^.

## Materials and methods

### Participants

This study included 50 female breast cancer patients who were getting treatments at Baheya Hospital in Cairo, Egypt, and 50 healthy individuals with matching ages as controls. After providing an explanation of the study each participant provided informed written consent. The study established an ethical approval from Baheya Research ethics committee (Registration Number 202311200051). All analyses were performed in accordance with the Declaration of Helsinki.

### Exclusion criteria

Participants with malignancies other than breast cancer, surgical interference of the intestines within the last 6 months, record of long use of antibiotics-continuous course of antibiotic therapy lasting longer than 2 weeks within the past 3 months-, infectious diarrhea, corticosteroids use within the last three months, nonsteroidal anti-inflammatory drug abuse prolonged use of NSAIDs exceeding the recommended dosage for more than 2 weeks without appropriate medical supervision-, autoimmune diseases, pregnancy, lactation, or substance addiction were excluded from the study.

### Collection, preservation, and transport of microbiota specimens

Fresh stool samples were collected from both the breast cancer patients and controls in sterile containers and transported immediately to the microbiology laboratory at Baheya Hospital. The samples were stored at − 20 °C until further processing.

### DNA extraction

Bacterial DNA was extracted from the stool samples using the Quick—DNA-™ Miniprep Plus Kit (Catalog number D4068, Zymo Research, USA) according to the manufacturer’s instructions.

### Primer sequences

Precise oligonucleotide primers were used to the 16 S rRNA gene sequences of *Lactobacillus*, *Bifidobacterium*, and *Streptococcus*. The primer sequences used in this study are listed in Table 1. The primers were commercially obtained from Willowfort (UK).

**Table 1:** Primers sequences of 16 S rRNA gene of *Bifidobacterium lactobacillus*, and *Streptococcus*.

Target bacteria		Primer sequence (5′–3′)	References
*Lactobacillus*	Forward	CAGCAGTAGGGAATCTTCCAC	^31^
	Reverse	GGCTTCTGGCACGTAGTTAG	
*Bifidobacterium*	Forward	CGGGTGAGTAATGCGTGACC	^32^
	Reverse	TGATAGGACGCGACCCCA	
*Streptococcus*	Forward	GCACTCGCTACTATTTCTTACCTCAA	^33^
	Reverse	GTCACAATGTCTTGGAAACCAGTAAT	

### SYBR green real-time PCR

HERA SYBR^®^ Green RT-qPCR Kit (HERA SYBR^®^ Green, Willowfort, UK) was used for amplification in a Thermocycler (DTLite, DNA Technology Research & Production, Russia). Ten microliters of the reaction mixture, consisting of 0.5 µl of forward and reverse primers, 1 µl of DNA extract, 5 µl of HERA SYBR^®^ Green Master Mix, and 3 µl of nuclease-free water, was used for the PCR amplification. The amplification protocol consisted of initial denaturation at 95 °C for 3 min, followed by 40 cycles of denaturation at 95 °C for 15 s, annealing at 60 °C for 30 s, and extension at 72 °C for 15 s. Standard curves were constructed using fourfold serial dilutions of DNA extracted from a pooled sample of healthy individuals.

### Metabolic syndrome data collection and diagnosis

Clinical parameters for diagnosing metabolic syndrome were obtained from patient records at Baheya Hospital. The diagnosis of metabolic syndrome was based on the criteria of American Heart Association/Updated NCEP ATP III (2004). A confirmed case of metabolic syndrome required the presence of three or more criteria, including, waist circumference exceeding 80 cm in females; fasting glucose levels of 100 mg/dL or higher; HDL cholesterol below 50 mg/dL in females; triglyceride levels of 150 mg/dL or higher; and blood pressure of 130/85 mmHg or higher.

### Statistical analysis

Version 26.0 of IBM SPSS software package (IBM Corp., USA) was used for data analysis. Qualitative data existed as percentage and frequency, and the test of Kolmogorov–Smirnov was used to check normality. The quantitative data were described using various statistical measures. The chi-square test was used to compare the variables between categories- breast cancer and metabolic syndrome-, while the Mann–Whitney U test and Kruskal–Wallis tests were used for not normally distributed quantitative variables.

## Results

### Clinical characteristics

The study included 50 female with non-metastatic invasive ductal carcinoma with a mean age of 62.3 years. Among them, 30 (60%) were diagnosed with metabolic syndrome, while 20 (40%) were considered metabolic syndrome-free. The control group consisted of 50 cases with no metabolic syndrome diagnosis.

### Breast cancer and metabolic syndrome

The prevalence of metabolic syndrome was higher in women with breast cancer (*p* < 0.001), indicating a strong association between metabolic syndrome and breast cancer.

### Comparison of bacterial counts

**Fig. 1:**
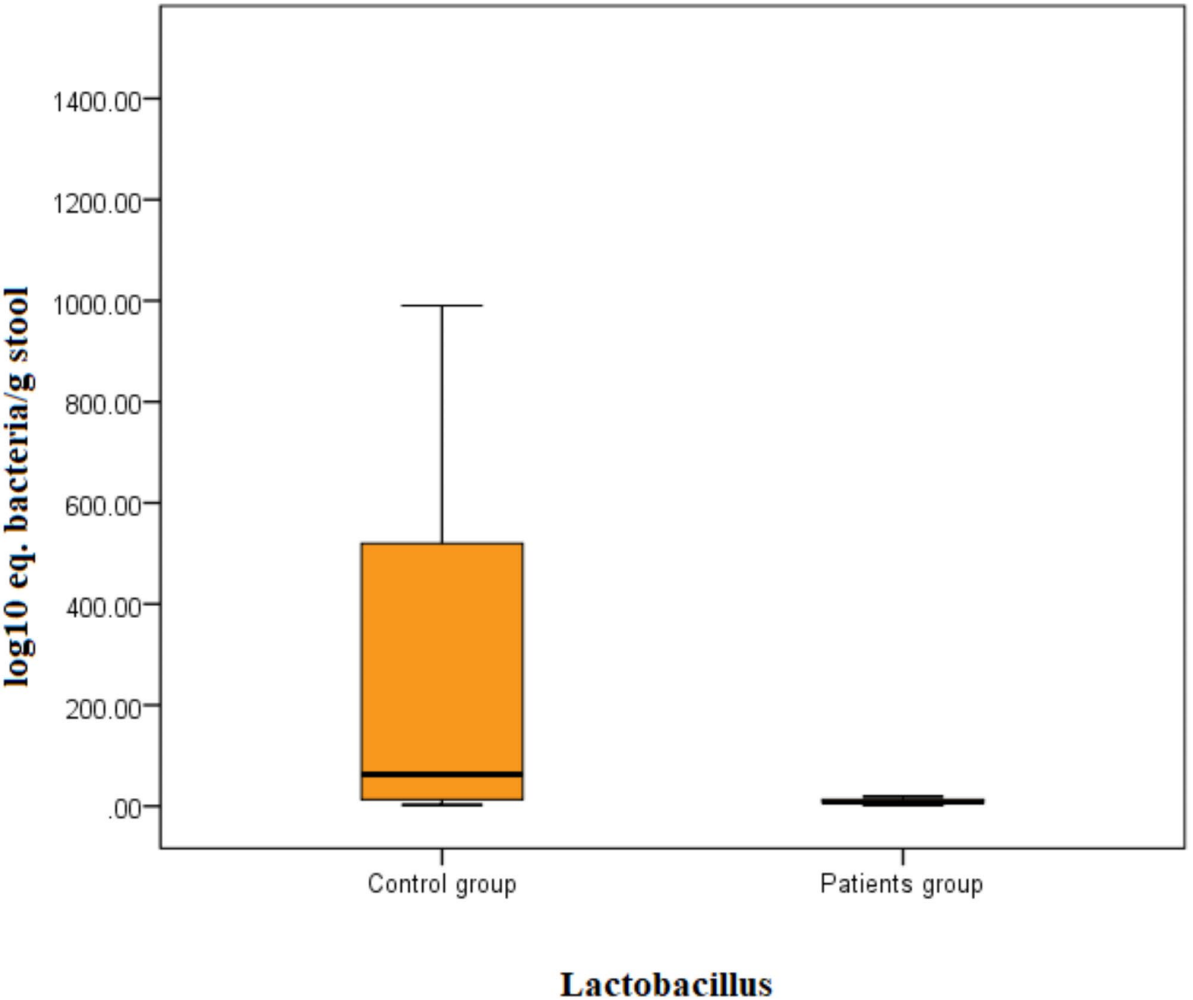
The loads of *Lactobacillus* bacteria in patients and control stools by the Mann–Whitney test. Bacterial group count expressed as log10 equivalent bacteria/g of feces.

**Fig. 2:**
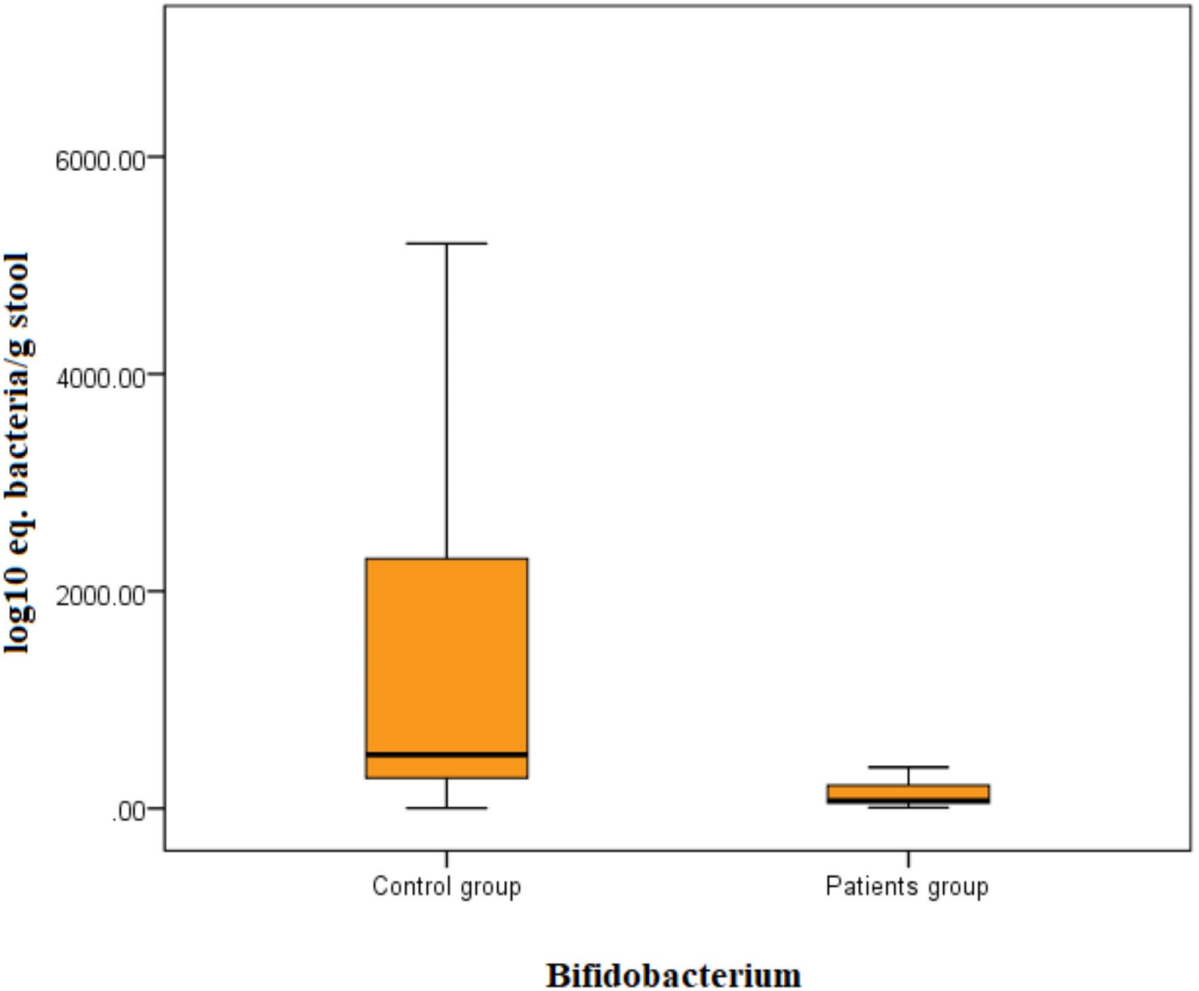
The load of *Bifidobacterium* bacteria in the stools of patients and control by the Mann–Whitney test. Bacterial group count expressed as log10 equivalent bacteria/g of feces.

**Fig. 3:**
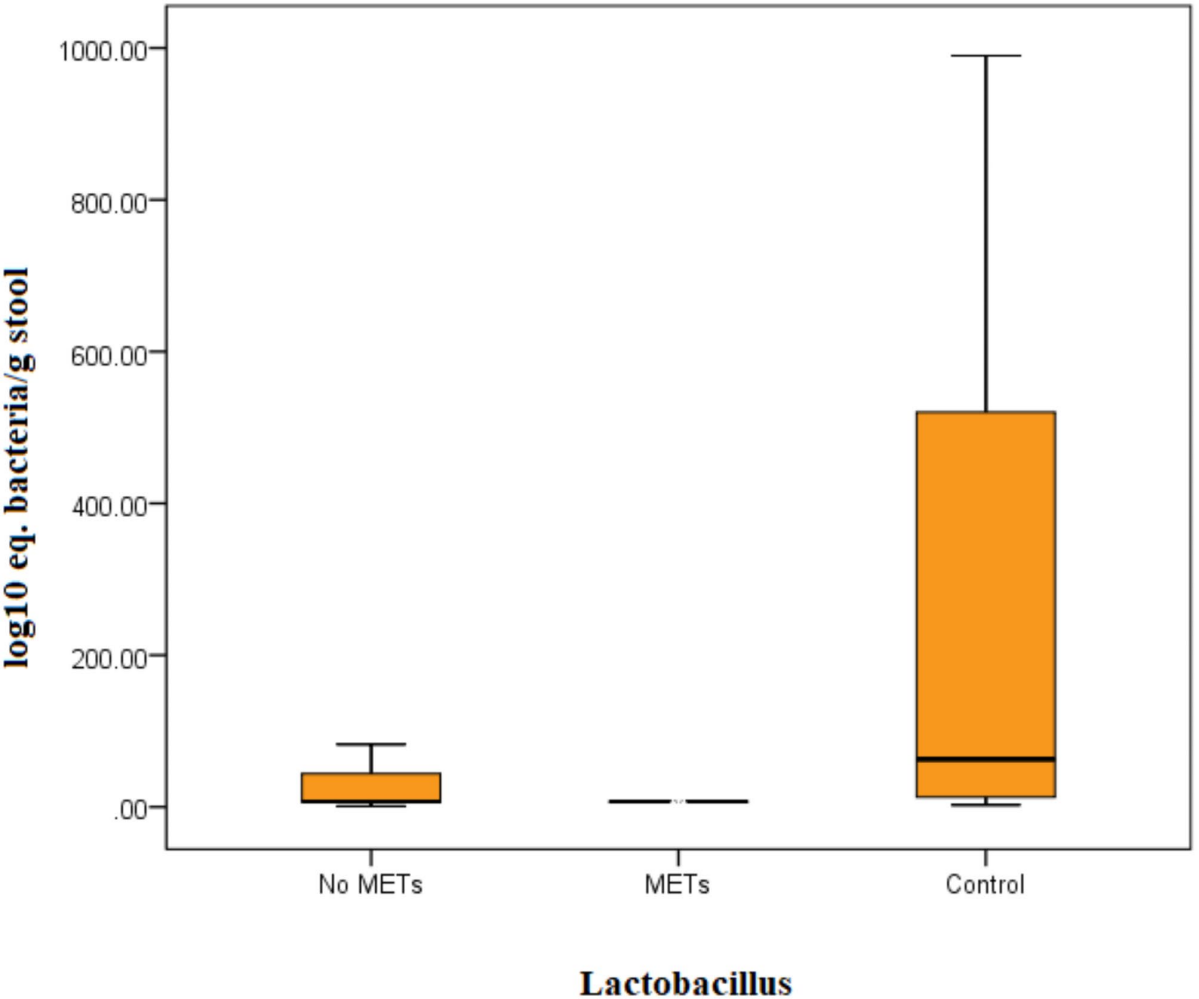
The load of *Lactobacillus* bacteria in the stools of separated patients regarding metabolic syndrome and control by Kruskall–Wallis test. Bacterial group count expressed as log10 equivalent bacteria/g of feces.

**Fig. 4:**
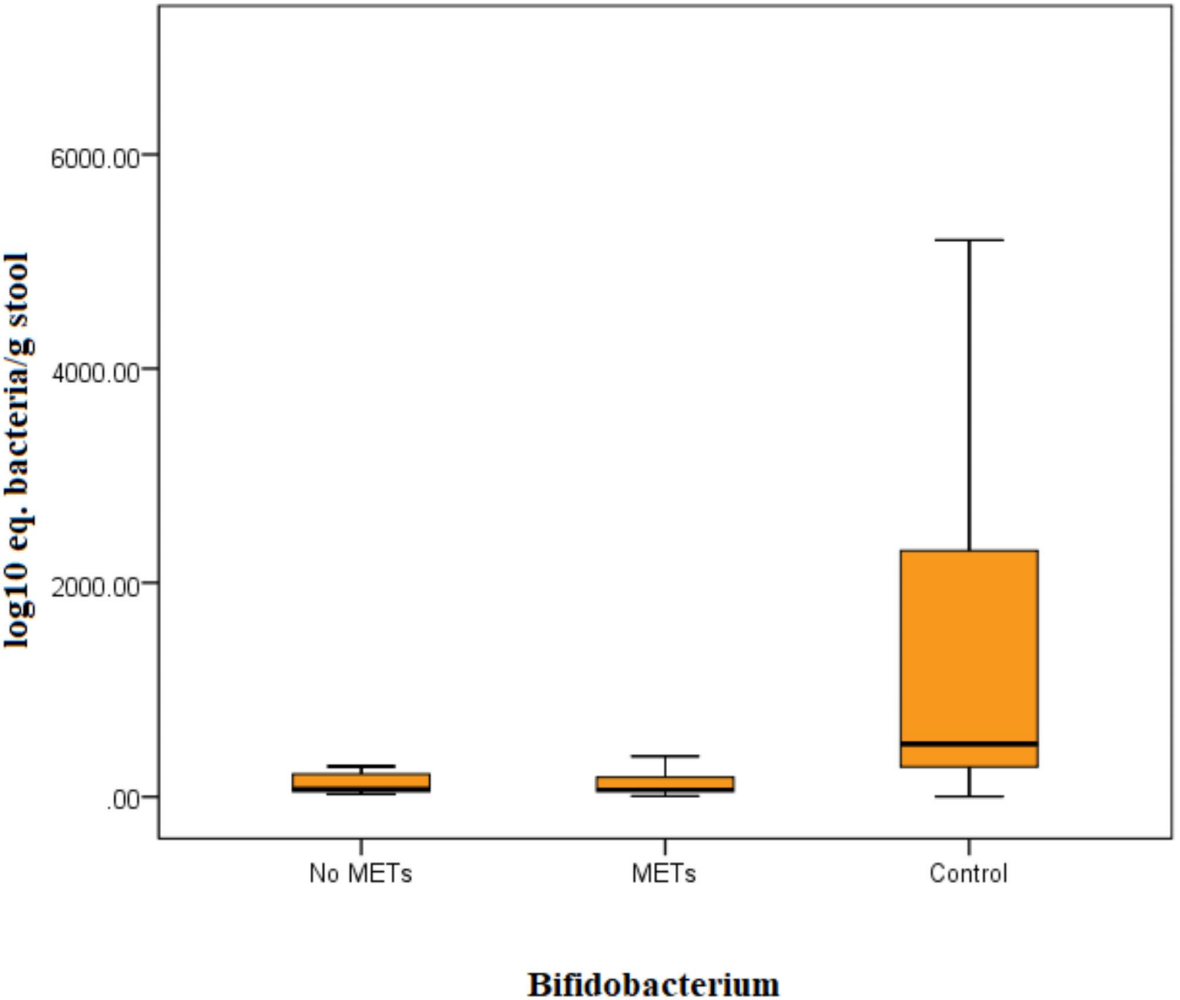
The load of *Bifidobacterium* bacteria in the stools of separated patients regarding metabolic syndrome and control by Kruskall–Wallis test. Bacterial group count expressed as log10 equivalent bacteria/g of feces.

Patients with breast cancer had significantly lower *Lactobacillus* (U = 4.7, *P* < 0.001) counts than controls (Fig. 1) and *Bifidobacterium* (U = 5.8, *P* < 0.001, Fig. 2). No significant difference was observed in *Streptococcus* counts (Table 2). There is no significant difference noticed in bacterial counts between breast cancer patients with and without metabolic syndrome. However, when compared separately with controls, there was a highly significant difference in *Bifidobacterium* and *Lactobacillus* counts for both groups of breast cancer patients (Figs. 3 and 4). No significant difference was detected in *Streptococcus* counts between the two groups (Table 3).

**Table 2:** Analysis contrasting the bacterial abundances in several groups.

		Control group	Patients group	Test value	*P*-value
Lactobacillus**	Median (IQR)	63 (13–520)	7.24 (7.18–13.1)	U = 4.709	< 0.001*
	Range	2.6–6200	1.13–430		
Bifidobacterium**	Median (IQR)	495 (280–2300)	71.39 (49.68–213.13)	U = 5.805	< 0.001*
	Range	2.9–6600	7.33–945		
Streptococcus**	Median (IQR)	0.26 (0.04–0.66)	0.16 (0.04–0.65)	U = 0.493	0.622
	Range	0–9.2	0–92		

The data were shown as the median (interquartile range; IQR); the Mann-Whitney U test was used to analyze the differences between the two groups.

*Statistically significant at *P* ≤ 0.05.

**Quantitative determination (log10 copies/g faeces).

**Table 3:** Comparison of the bacterial abundance between patients with and without MetS.

		No METs	METs	Test value	*P*-value
*Lactobacillus*	Median (IQR)	7.26 (7.19–43.84)	7.24 (6.46–7.3)	0.772	0.440
	Range	1.13–82.57	2.56–430		
*Bifidobacterium*	Median (IQR)	73.51 (50.21–213.13)	68.74 (49.68–183.33)	0.376	0.707
	Range	24.26–588	7.33–945		
*Streptococcus*	Median (IQR)	0.16 (0.04–1.38)	0.16 (0.04–0.58)	0.317	0.751
	Range	0–9.9	0–92		

The data were shown as the median (interquartile range; IQR); the Mann–Whitney U test was used to analyze the differences between the two groups.

*Statistically significant at *P* ≤ 0.05.

**Quantitative determination (log10 copies/g faeces).

The study conducted a ROC analysis and determined that the optimal cut-off points for *Bifidobacterium* and *Lactobacillus* were 213.13 log10 cells/g and 7.31 log10 cells/g, respectively. Breast cancer patients had a significantly higher frequency of falling below the cut-off point for *Bifidobacterium* (odds ratio 10.63, 95% CI 4.146–27.252, *P* = 0.0001) and *Lactobacillus* (odds ratio 26.000, 95% CI 8.793–76.876, *P* = 0.000) compared to controls.

## Discussion

Breast cancer is a widespread malignancy in women, associated with various environmental and hormonal factors. Lifestyle choices, such as high-fat diets and reduced physical activity, are linked to metabolic syndrome (MetS), which plays a role in breast cancer beginning. Previous studies have proven that women with MetS have a higher probability of breast cancer^34,35^.

The gut microbiota has been the subject of many research in recent years, with disturbances in this ecosystem suspected to be involved in various diseases^36^. It has been discovered that individuals with breast cancer have considerably changed microbiota makeup, with fewer varied gut microorganisms^37,38^. This study is the first in Egypt to link intestinal microbiota with breast cancer patients.

The study’s findings of increased breast cancer risk in women with MetS are consistent with previous research^39,40^. The relationship between MetS and breast cancer is not fully understood, but the role of microbiota may play a part. Intestinal microbiota has numerous roles in human pathology physiology and, including contributing to host nutrition, energy harvesting, fermentation of food components, and vitamin production^41–43^. It also improves the immune system, homeostasis of intestinal epithelial, and immunity against pathogens^44–47^.

Human and animal studies have linked the gut microbiota to metabolic syndrome. An imbalance between intestinal microbes and the host immune system can cause bacterial fragments to enter the bloodstream through the intestine barrier, leading to systemic inflammation. This inflammation can lead to insulin resistance, diabetes, and obesity^48,49^.

The current study found that breast cancer patients had significant dysbiosis, with a decline in Lactobacillus and Bifidobacterium counts. These bacterial groups are associated with a reduction in fecal bacterial enzymes, including ß-glucuronidase, which deconjugates estrogens that could contribute to breast cancer risk^50,51^. Gut bacteria, such as Lactobacillus, Bifidobacterium, and Streptococcus, are known to inhibit the production of pro-inflammatory cytokine TNF-α, which is associated with breast cancer growth and metastasis^52,53^. These bacterial groups are also engaged in the synthesis of the anti-inflammatory short-chain fatty acids (SCFAs), and support intestinal epithelial cells^54,55^.

Correlations between specific microbial taxa and breast cancer underscore the role of dysbiosis in disease development. For instance, studies show increased relative abundances of *Bacillus*, *Enterobacteriaceae*, and *Staphylococcus* in adjacent tissue of breast cancer patients compared to controls, suggesting microbial modulation of the local immune microenvironment^56^. Moreover, the presence of anti-inflammatory strains such as *Faecalibacterium prausnitzii* has been linked to protective effects against inflammatory diseases, whereas its reduced abundance correlates with higher inflammation and potential cancer risk^57^. These associations are further supported by mechanisms where short-chain fatty acids (SCFAs) like butyrate exert anti-tumorigenic effects by inhibiting histone deacetylases (HDACs), leading to histone hyperacetylation, altered gene expression, and tumor suppression^58^. While causality is established in certain pathways, such as estrogen reabsorption, other findings remain associative, necessitating further research to determine direct mechanistic links between gut microbiota and breast cancer.

The study highlights the need for further investigation into the gut microbiota’s role in breast cancer, particularly in the gut microbiota, which could be critical in understanding factors associated with breast cancer. The exploration of the fecal microbiota only reflects the microbiota present in the upper intestine, and there may be notable variations in the composition of gut microbiota depending on the gut segment. Thus, additional investigations on other bacterial role players are required to fully understand the gut microbiota’s role in breast cancer.

Public health initiatives targeting metabolic syndrome—a cluster of conditions including obesity, hypertension, high blood sugar, and abnormal cholesterol levels—are crucial in reducing breast cancer incidence and mortality. The Women’s Health Initiative randomized trial demonstrated that dietary interventions, particularly a low-fat diet, significantly decreased breast cancer mortality, especially among women with multiple metabolic syndrome components^59^. Another study focusing on African American women demonstrated that programs led by lay health coaches effectively reduced MetS prevalence by promoting healthier lifestyles within the community^60^. Additionally, digital health interventions have shown promise in combating MetS. The e-Motivate4Change program, an online platform, successfully improved participants’ health-related lifestyle scores and self-efficacy, leading to significant reductions in body mass index (BMI) and cholesterol levels. This suggests that technology-driven approaches can be valuable tools in public health strategies aimed at preventing and managing MetS^61^.

## Conclusion

The study found significant differences in Lactobacillus and Bifidobacterium counts between breast cancer patients and controls, suggesting a possible influence on the development of breast cancer. However, the study’s limitations include the analysis of only total counts for certain bacterial groups and the examination of the gut microbiota through stool samples, which may not fully reflect the gut microbiota’s composition.

## Data Availability

The datasets used and/or analyzed during the current study are available from the corresponding author on reasonable request.

## Abbreviations

MetS: Metabolic syndrome
HDL: high-density lipoprotein
GI: gastrointestinal tract
TNF-α: tumor necrosis factor-alpha
SCFAs: Short-chain fatty acids
